# Genome Analysis of Two *Arthrobacter* Bacteriophages, Andrew and RedFox, from Subcluster AS3 Isolated from Soil in New York and Pennsylvania

**DOI:** 10.17912/micropub.biology.002367

**Published:** 2026-09-29

**Authors:** Paige Baldwin, Megan Dennis, Niran Isik, Hanan Isik, Deborah Jacobs-Sera, Juliana Magna, Mia Mazakas, Gabriella Rice, Sarah Swerdlow, Megan Ulbrich, Matthew Farber, Marina Bogush

**Affiliations:** 1 Biological & Biomedical Sciences, Rowan University, Glassboro, NJ, United States; 2 Biology, Marist University, Town of Poughkeepsie, NY, United States; 3 Biological Sciences, University of Pittsburgh, Pittsburgh, PA, United States; 4 Biological Sciences, University of Pittsburgh at Greensburg, Greensburg, PA, United States

## Abstract

Novel bacteriophages Andrew and RedFox were isolated from soil samples collected in New York and Pennsylvania and propagated using *Arthrobacter globiformis* B-2979. Both phages are assigned to actinobacteriophage subcluster AS3 based on gene content. Although these phages exhibit conserved genomic content characteristics of the AS3 subcluster, there are notable differences. In particular, phage Andrew gene *25* in the central region of the genome and genes *64, 65,* and *66* near the end of the genome lack homologs in cluster AS3 or in the actinobacteriophage database more broadly.

**Table 1. Bacteriophages Andrew and RedFox:** Sequencing data and genome characteristics

**Table:** 

Genome Characteristics	Andrew	RedFox
Subcluster	AS3	AS3
Host Bacterium	Arthrobacter globiformis B-2979	Arthrobacter globiformis B-2979
Soil Sample Coordinates	40.445472 N, 79.998708 W	41.7203 N, 73.9356 W
Plaque morphology	Clear	Clear
Number of reads	685,575	366,290
Sequencing Coverage, fold	1087	1400
Predicted Cluster Life Cycle	Temperate	Temperate
Genome size (bp)	38,802	38,728
Genome termini	3'single-stranded overhang	3'single-stranded overhang
	5'-GAGTTGCCGGCA	5'-GAGTTGCCGGCA
GC% Content	65.5	66.1
No. of predicted genes	72	71
No. of predicted genes with putative functions assigned	36	40
No. of tRNAs	0	0
GenBank accession #	MH834595	OR195049
SRA accession #	SRX31241826	SRX201657

## Description

Bacteriophages provide a means to modulate bacterial populations and therefore have practical applications in medicine and industry, including the treatment of bacterial infections (Hibstu et al. 2022; Hatfull et al. 2022) and the preservation of food (Imran et al. 2023). Given their abundance and diversity, the isolation and characterization of novel bacteriophages promise to advance their application. Here, we described the isolation and characterization of two bacteriophages using *Arthrobacter globiformis* , a soil bacterium that is both widely distributed and ecologically significant (Klyczek et al., 2018).

Bacteriophages Andrew and RedFox were isolated by enrichment (Zorawik et al. 2024) from soil samples collected in mildly wet and nutrient-rich environments in Pittsburgh, Pennsylvania (GPS coordinate: 40.445472 N, 79.998708 W) and Poughkeepsie, New York (GPS coordinate: 41.7203 N, 73.9356 W), respectively. Andrew and RedFox soil samples were suspended in peptone-yeast extract-calcium (PYCa) medium and shaken at 250 rpm for 1 and 1.5 hours, respectively. The suspensions were centrifuged, filtered through a 0.22-μm filter, and the filtrate inoculated with *Arthrobacter globiformis B-2979* for 48 hours at 30°C. The enriched cultures were then filtered again, and 10 μL of each filtrate was plated in PYCa top agar with *A. globiformis* and incubated at 30°C for 48 hours. Phages were purified through three successive rounds of plating for isolated plaques. Both Andrew and RedFox produced clear plaques.

Transmission electron microscopy with 1% uranyl acetate staining for Andrew or UranyLess staining for RedFox revealed siphovirus morphologies characterized by icosahedral capsids and flexible tails. The corresponding plaque and transmission electron microscopy (TEM) images are available through the PhagesDB entries for Andrew and RedFox, respectively (Russell and Hatfull 2017). DNA was extracted from bacteriophage lysates using the Promega DNA Wizard Cleanup Kit. Sequencing libraries were prepared using the NEB Ultra II FS DNA library kit and run on the Illumina MiSeq platform (v3 reagents), yielding 150-base single-end reads that were assembled into genomes using Newbler and Consed software (Russell 2018). Sequencing data and genome characteristics are presented in Table 1.

Bacteriophage genome annotations were completed using DNA Master v5.23.6 (https://phagesdb.org/DNAMaster/) embedded with Glimmer v3.02 (Delcher et al. 2007) and GeneMark v2.5p (Besemer and Borodovsky 2005). Gene start sites were refined using Starterator v1.2 (http://phages.wustl.edu/starterator/). To identify putative functions of protein-coding genes, several comparative tools were used, all with default parameters for all software. These included Phamerator (Cresawn et al. 2011) with the Actino_draft database v578; BLAST, using the Actinobacteriophage and NCBI non-redundant databases (Altschul et al. 1990); and HHPRED, using the PDB_mmCIF70, Pfam v.36, and NCBI Conserved Domains databases (Söding et al. 2005). Aragorn v1.2.41 (Laslett and Canback 2004) or tRNAscanSE v.2.0 (Lowe and Chan 2016) were used to identify tRNAs.

Andrew has a genome of 38,802 bp, with a GC content of 65.5%. RedFox is 38728 bp long, with a 66.1% GC content. Both have 3′ 12-base single-stranded sticky ends. The GC content is typical of other *Arthrobacter* bacteriophages, which range from 45.1% to 68.5% (Klyczek et al. 2018; Russell and Hatfull 2017). These two bacteriophages were sorted into cluster AS and subcluster AS3 based on gene content similarity (GCS) of at least 35% to bacteriophages in the Actinobacteriophage database (Russell and Hatfull 2017; Pope et al. 2017).

Genome annotation revealed that Andrew contains 72 predicted genes, 36 of which were assigned putative functions, whereas RedFox contains 71 predicted genes, 40 of which were assigned putative functions (Table 1). Neither genome was found to contain tRNAs genes (Table 1). Both Andrew and RedFox demonstrated high GCS of 83.1% and shared highly conserved genome architectures characteristic of the AS3 subcluster (Glaser et al. 2026). Both genomes displayed a modular structure containing conserved regions associated with DNA packaging, virion structure and assembly, lysogeny, and DNA replication (Glaser et al. 2026). Despite this overall conservation, several regions display greater genomic divergence when compared with AS3 cluster phages, including gene *25* in the central region of the Andrew genome and genes *64* , *65* and *66* near the end of Andrew genome. These genes lack homologs in the actinobacteriophage database, to date, adding to the diversity of gene sequences uncovered for actinobacteriophages through the isolation and characterization of novel phages.

GenBank accession numbers for Andrew and RedFox are MH834595 and OR195049). SRA accession numbers for Andrew and RedFox are SRX31241826 and SRX20165779 .
